# Prolonged Environmental Enrichment Promotes Developmental Myelination

**DOI:** 10.3389/fcell.2021.665409

**Published:** 2021-04-26

**Authors:** Evan Z. Goldstein, Vera Pertsovskaya, Thomas A. Forbes, Jeffrey L. Dupree, Vittorio Gallo

**Affiliations:** ^1^Center for Neuroscience Research, Children’s Research Institute, Children’s National Hospital, Washington, DC, United States; ^2^School of Medicine and Health Sciences, The George Washington University, Washington, DC, United States; ^3^Department of Anatomy and Neurobiology, Virginia Commonwealth University, Richmond, VA, United States

**Keywords:** enriched environment, oligodendrocyte, myelin, hypermyelination, glia, development, motor coordination

## Abstract

Postnatal neurodevelopment is profoundly influenced by environmental experiences. Environmental enrichment is a commonly used experimental paradigm that has uncovered numerous examples of experience-dependent plasticity in health and disease. However, the role of environmental enrichment in normal development, especially glial development, is largely unexplored. Oligodendrocytes, the myelin-forming glia in the central nervous system, provide metabolic support to axons and establish efficient saltatory conduction by producing myelin. Indeed, alterations in myelin are strongly correlated with sensory, cognitive, and motor function. The timing of developmental myelination is uniquely positioned to be influenced by environmental stimuli, as peak myelination occurs postnatally and continues into adulthood. To determine if developmental myelination is impacted by environmental experience, mice were housed in an enriched environment during peak myelination through early adulthood. Using translating ribosome affinity purification, oligodendrocyte-specific RNAs were isolated from subcortical white matter at various postnatal ages. RNA-sequencing revealed that differences in the oligodendrocyte translatome were predominantly evident after prolonged and continuous environmental enrichment. These translational changes corresponded with altered oligodendrocyte lineage cell dynamics and enhanced myelination. Furthermore, consistent with increased developmental myelination, enriched mice displayed enhanced motor coordination on a beam walking task. These findings indicate that protracted environmental stimulation is sufficient to modulate developmental myelination and to promote behavioral function.

## Introduction

Environmental experiences exert significant influence onto the complex genetic programs that drive neurodevelopment. Seminal work using monocular deprivation highlights the impact of experiential stimuli on postnatal neurodevelopment (Wiesel and Hubel, 1963). For over 50 years, environmental enrichment (EE) —an experimental paradigm that exposes animals to increased social interactions, novel stimuli, and voluntary exercise—has been used to study the role of experiential stimuli in brain health and disease (Diamond et al., 1964; van Praag et al., 2000). EE promotes cellular, anatomical, and functional improvements under non-pathological conditions—such as learning and memory (Nilsson et al., 1999; Kobayashi et al., 2002) —and in numerous models of neurological disorders, including spinal cord injury, stroke, and Alzheimer’s disease (Johansson, 1996; Lazarov et al., 2005; Hutson et al., 2019). While neurological effects of EE are well-characterized in the adult brain, the impact of EE on the developing brain has not been widely explored outside of the visual system (Cancedda et al., 2004). Furthermore, EE research has primarily focused on neuronal and synaptic changes, despite critical roles of glial cells in neuroplasticity.

To begin to address this gap in understanding, we recently showed that continuous EE during a critical period of intervention was sufficient to ameliorate subcortical white matter (WM) dysmyelination and recover motor deficits caused by perinatal hypoxic brain injury (Forbes et al., 2020). This recovery was associated with complex cellular and molecular changes in oligodendrocytes (OLs) —the myelinating glia of the central nervous system (CNS). While this work demonstrated the utility of EE for recovery from neurodevelopmental injury, it is still unknown if EE can impact normal developmental myelination. Indeed, myelination is uniquely positioned for significant experience-dependent influence during development, as it peaks early postnatally and continues throughout adulthood (Semple et al., 2013). Furthermore, growing evidence indicates that myelination is modulated by activity, learning, memory, and social interactions (Liu et al., 2012; Makinodan et al., 2012; Gibson et al., 2014; McKenzie et al., 2014; Mitew et al., 2018; Pan et al., 2020).

Therefore, we investigated whether EE was sufficient to influence subcortical WM development. Beginning at postnatal day (P) 15, mice were continuously housed with 8–12 cage mates with free access to a running wheel and toys that were changed and rearranged regularly. Utilizing OL-specific RNA-sequencing, we show—for the first time—that protracted periods of EE are required for substantial molecular changes to normally developing OLs. After additional time spent in EE, these molecular changes translated into altered OL lineage cell dynamics, enhanced myelination, and improvement in subcortical WM-dependent locomotor coordination. Overall, these findings indicate that normal developmental myelination is susceptible to experience-dependent adaptation, thereby providing further evidence of myelin plasticity in the CNS.

## Methods

### Animals

All animal procedures were conducted in compliance with the Institutional Animal Care and Use Committee (IACUC) of the Children’s National Hospital (protocol #30473) and the *Guide for the Care and Use of Laboratory Animals* (National Institutes of Health). Male and female mice were used in all experiments. All mouse cages were individually ventilated and pups were weaned at P21. CNP-bacTRAP (The Jackson Laboratory #009159) mice were used for OL-specific RNA-seq experiments and C57BL/6J (The Jackson Laboratory #000664) mice were used for all other experiments.

### Enriched Environment

At P15, litters were randomly selected to either be housed in an enriched environment or serve as controls housed in standard environment cages. The enriched environment protocol consisted of cages assembled from clear Plexiglass (24 cm W x 20 cm H x 46 cm L) with continuous access to a running wheel, allowing for voluntary physical activity. Additionally, the cages were outfitted with various objects with a wide range of textures (wooden blocks, plastic “habit-trails” metal lofts, and balls). These objects were changed and repositioned every 3–4 days throughout the environmental enrichment period in order to establish novelty. The standard environment protocol consisted of smaller cages (16 cm W × 13 cm H × 37 cm L) assembled from clear Plexiglass that did not have any additional objects. Mice housed in an enriched environment were reared in larger groups (*n* = 8–12 animals per cage) during periods of enrichment, while control mice were reared in smaller groups (*n* = 2–5 animals per cage). Both groups of mice were provided nesting materials.

### Translating Ribosome Affinity Purification and RNA-Seq

At the indicated time points, OL RNAs were isolated from CNP-bacTRAP using a previously described protocol (Heiman et al., 2014; Forbes et al., 2020). Subcortical WM was rapidly dissected in cold dissection buffer (2.5 mM HEPES, 35 mM glucose and 4 mM NaHCO3, and 100 μg/ml cycloheximide in HBSS) and homogenized in lysis buffer over ice (20 mM HEPES, 150 mM KCl and 10 mM MgCl2, 0.5 mM DTT, 100 μg/ml cycloheximide, 10 μl/ml rRNasin, 10 μl/ml Superasin, and EDTA-free protease inhibitors in RNase-free water). Following centrifugation at 1,000 × g for 10 min, 1% NP-40 and 1% DHPC were added to supernatants, incubated on ice for 5 min, and centrifuged at 10,000 × g for 10 min. Supernatants were mixed with GFP antibodies (HtzGFP-19F7 and HtzGFP-19C8, Memorial Sloan Kettering Center) bound to Streptavidin MyOne T1 Dynabeads (Invitrogen) overnight at 4°C. A high salt buffer (20 mM HEPES, 350 mM KCl, 10 mM MgCl2, 1% NP-40, 0.5 mM DTT, and 100 μg/ml cycloheximide in RNase-free water) was used to wash beads 4 times, and RNA was isolated using the Absolutely RNA Nanoprep Kit (Agilent).

Libraries were prepared from RNA (RIN > 7, Agilent) using the Trio RNA-Seq Library Preparation Kit (NuGen), according to the manufacturer’s protocol. 50 base pair (bp), paired-end reads were obtained from the NovaSeq 6000 System (Illumina). The first 5 bp were trimmed from the 5′ end of the reads using Trimmomatic (Bolger et al., 2014), as recommended by the library preparation kit. Reads were mapped to the mouse reference genome (Genome Reference Consortium Mouse Build 38—mm10) using STAR (Dobin et al., 2013), and read counts were generated using HTseq (Anders et al., 2015). Differential gene expression and normalized read counts were generated using DESeq2 package for R (Love et al., 2014). Reported DEGs had normalized read counts above 5 in all samples, and an adjusted *p*-value < 0.05 (Wald test with Benjamini-Hochberg *post hoc*). Gene ontological analysis was performed using g:Profiler software (Raudvere et al., 2019). Ingenuity Pathway Analysis (Qiagen) was used to identify cellular and molecular functions, pathways and upstream regulators implicated from differentially expressed genes between EE and Standard groups.

### Tissue Processing and Immunohistochemistry

For the immunohistochemical analysis, mice were anesthetized with isoflurane and transcardially perfused with 0.1 M phosphate buffered saline (PBS), followed by 4% paraformaldehyde (PFA). Brains were post-fixed in 4% PFA overnight, coronally sectioned on a freezing microtome at 40 μm, and stored at 4°C in 0.1M PBS with 0.01% sodium azide.

Immunohistochemistry was performed on free-floating sections that were blocked in solution containing PBST (0.1% TritonX-100) and 20% normal donkey serum (NDS) for 1h and incubated overnight at 4°C in primary antibodies diluted in PBST and 5% NDS. Primary antibodies include: mouse anti-CC1 (Millipore; 1:250), rabbit anti-NG2 (Millipore; 1:250), rabbit anti-Olig2 (Millipore; 1:500), mouse anti-Olig2 (Millipore; 1:250), rat anti-Ki67 (eBioscience; 1:500), rabbit anti-myelin basic protein (Abcam; 1:250), and rabbit anti-IBA1 (Wako; 1:250). Sections were incubated in species-appropriate secondary antibodies (Jackson Immunoresearch; 1:500) for 2h at room temperature. Sections were placed on slides and mounted with DAPI Fluoromount-G (Southern Biotech).

### Image Acquisition and Analysis

A Leica TCS SP8 confocal microscope was used to image tissue. Z-stack images of 1 μm thick planes were collected using LAS X (Leica). Images were viewed using ImageJ (NIH). All histological quantifications were performed in a blinded manner. Using the ImageJ “Cell Counter” plug-in, immunolabeled cells were manually counted in each image. OPCs were only counted when 75+ % of an OLIG2^+^ nucleus was surrounded by NG2^+^ staining. Six bilateral images were taken from each section of the subcortical WM, including two from the corpus callosum, two from the cingulum, and two from the external capsule. For each image, the total number of cells was counted and normalized to volume. The percentage of IBA1-expressing area was calculated using ImageJ. Each value presented represents the average IBA1 expression from six bilateral images taken in the subcortical WM. To quantify the subcortical WM area, myelin basic protein (MBP) expression in the MW was outlined and measured using ImageJ.

### Electron Microscopy

Deeply anesthetized mice were transcardially perfused with a 4% paraformaldehyde and 5% glutaraldehyde in 0.1 M Millonig’s phosphate buffer. After 2 weeks of post-fixation, brains were cut into 1 mm sagittal sections using a brain matrix, thoroughly rinsed in 0.1 M cacodylate buffer, post-fixed in 2% osmium tetroxide, rinsed in 0.1M cacodylate buffer, dehydrated in serial dilutions of ethanol, and embedded in Polybed 812 resin (PolySciences, Warrington, PA). Sections (1 μm) were stained with toluidine blue and used to identify the body of the corpus callosum at the level of the fornix. 90 nm sections of this area were stained with uranyl acetate and lead citrate. Sagittal sections of corpus callosum were inspected with a JEM 1400 plus transmission electron microscope (JEOL) and imaged with a One View 4K CCD camera (Gatan Inc.) housed in the Department of Anatomy and Neurobiology Microscopy and Imaging Facility at Virginia Commonwealth University. For each sample, 12–13 electron micrographs were collected from the corpus callosum and used to quantify myelinated axons. Micrographs were viewed and analyzed with ImageJ (NIH). Myelin thickness was reported as a g-ratio. This value represents the axonal diameter divided by the diameter of the axon plus the myelin. Thus, a lower g-ratio value is indicative of an axon with thicker myelin. For each myelinated axon, two axon diameters and two myelin widths were measured. Myelin thickness was computed from the average of the four radial measurements per sheath, avoiding regions of fixation artifact or tongue processes. Axons that had diameters less than 0.3 μm (characteristic of unmyelinated fibers) were not included in the analysis. Measurements were collected blinded to experimental groups, and at least 100 axons were measured per mouse.

### Inclined Beam Task

The examiner was blinded to groups for each behavioral experiment that was performed. All of the behavioral studies were conducted using naïve mice that had not previously been exposed to experimental testing. The inclined beam-walking task involved two elevated 80-cm long wooden beams at a 30° angle. One of the beams was 1-cm wide and the other was 2-cm wide. At the top of each inclined beam, a dark box with animal bedding was placed as a target for the mice to reach. The beams were cleaned between each mouse with 30% ethanol. As the mice traversed the inclined beam, the number of foot slips was recorded and averaged from four trials. Behavioral experiments were performed at P60 or P90.

### Statistics

The number of animals used for each experiment is indicated in each figure or figure legend. Statistical significance was calculated with GraphPad Prism 8.4.3 software. All data is depicted as averages ± SEM. All cellular, anatomical, and behavioral data were statistically compared using unpaired t-tests to examine whether differences are present between experimental groups. To determine statistical significance, a two-tailed type 1 error (*p* value < 0.05) was used. Exact *p* values are provided for comparisons reaching statistical significance in the figures.

## Results

### Prolonged EE Alters the OL Translatome in the Subcortical WM

**FIGURE 1:**
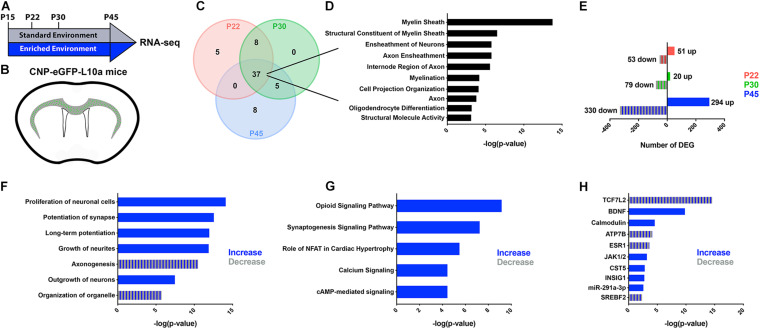
RNA-seq reveals changes in the OL translatome following prolonged EE. **(A,B)** Schematic representation of the experimental timeline **(A)** and GFP-expressing oligodendrocytes in the subcortical WM of CNP-bacTRAP mice **(B)**. **(C)** Venn Diagram of the 50 highest-expressed RNAs in subcortical WM OLs at P22 (red), P30 (green), and P45 (blue). **(D)** Gene ontological analysis of 37 OL RNAs highly expressed at all timepoints. This list was generated using g:profiler, and is comprised of the top ten molecular functions, biological processes, or cellular components ranked by −log(*p*-value). **(E)** Number of differentially expressed genes between EE and SD mice at P22, P30, and P45 (Wald test with Benjamini-Hochberg *post hoc*, adjusted *p* < 0.05, normalized counts > 5) (P22: SD, *n* = 4, EE, *n* = 6; P30: SD, *n* = 5, EE, *n* = 4; P45: SD, *n* = 4, EE, *n* = 6). **(F–H)** Predicted increases (blue) and decreases (blue/gray stripes) in cell and molecular functions **(F)**, pathways **(G)**, and upstream regulators **(H)**, in EE compared to SD OLs at P45 (determined by directional z-scores). Cell and molecular functions, pathways, and upstream regulators are ranked based on *p-*value as determined using Fisher’s Exact Test.

We previously established that EE induced significant cellular and molecular changes in subcortical WM OLs during a critical period after neonatal hypoxia (Forbes et al., 2020). To determine if EE impacts normal developmental myelination at a molecular level, we combined translating ribosome affinity purification (TRAP) with RNA sequencing on CNP-bacTRAP mice. Following polysome stabilization, actively translating RNAs were immunoprecipitated from post-mitotic OLs based on enhanced green fluorescent protein (GFP) expression in the R10a ribosomal subunit (Heiman et al., 2014). At various postnatal timepoints—P22, P30, and P45—OL RNAs isolated from the subcortical WM were sequenced from mice housed in either a Standard (SD) or Enriched Environment (EE) (Figures 1A,B). To confirm the validity of this technique, the 50 most abundant RNAs were compared at each time point (Figure 1C and Supplementary Tables 1–3). Gene ontological analysis of the 37 RNAs highly expressed at every time point confirmed that the TRAP technique isolated RNAs from OLs (Figure 1D). We next compared the translatomes of EE and SD OLs by quantifying the number of differentially expressed genes (DEGs) (Figure 1E). After 7 days in EE (P15-P22), 104 genes were differentially expressed (51 up-regulated and 53 down-regulated) in subcortical WM OLs (Figure 1E and Supplementary Table 4). Similarly, 99 DEGs were identified (20 up and 79 down) after 15 days of EE (P15-P30) (Figure 1E and Supplementary Table 5). Interestingly, after continuous EE from P15 to P45, there was more than a sixfold increase in the number of DEGs in subcortical WM OLs (626 DEGs; 294 up and 330 down), suggestive of substantial molecular changes in subcortical OLs following 30 days of EE (Figure 1E and Supplementary Table 6).

To better define the relevance of the changes observed in the OL translatome after continuous EE from P15 to P45, we used Ingenuity Pathway Analysis (IPA; Qiagen)—software that provides biological context to lists of DEGs. At P45, IPA analysis predicted an increase of various cell and molecular processes related to synapse potentiation and cellular outgrowth (Figure 1F). Similarly, synaptogenesis and calcium signaling pathways were predicted to be increased after EE from P15-P45 (Figure 1G). In the context of OLs, these predicted changes in function and signaling align with cells that are extending more myelin sheaths. Indeed, synaptic proteins are expressed in OLs, and disruption of their expression results in abnormal myelination (Hughes and Appel, 2019). Additionally, the calcium/calmodulin-dependent kinase type IIβ (CaMKIIβ) is an actin cytoskeleton protein that regulates myelin thickness (Waggener et al., 2013). Further supporting these predicted functional changes in OLs, calmodulin was identified as an upstream regulator of many of the DEGs at P45, along with other regulators known to effect OLs and their maturation (TCF7L2, BDNF, JAK1/2) (Figure 1H). Altogether, these data indicate that continuous EE for 30 days causes molecular changes in subcortical WM suggestive of enhanced myelination.

### Prolonged EE Increases Corpus Callosum Myelination

**FIGURE 2:**
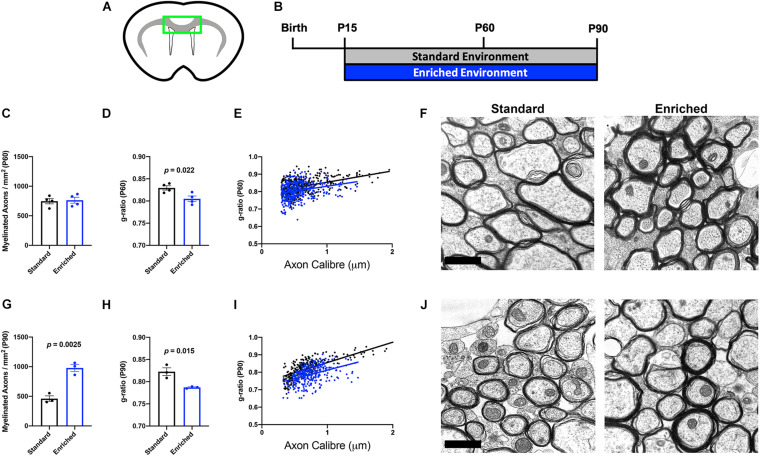
Prolonged EE increases corpus callosum myelination. **(A)** Schematic depiction of corpus callosum (green box) used for EM analysis. **(B)** Experimental timeline. **(C,G)** Number of myelinated axons in the corpus callosum of SD (black) and EE (blue) mice at P60 **(C)** and P90 **(G)**. **(D,H)** Average g-ratio of myelinated axons in the corpus callosum of SD (black) and EE (blue) mice at P60 **(D)** and P90 **(H)**. **(E,I)** Scatter plots depicting the g-ratios of individual corpus callosum axons relative to axon diameters in SD (black) vs. EE (blue) at P60 **(E)** and P90 **(I)**. **(F,J)** Representative electron micrographs from P60 **(F)** and P90 **(J)**. Scale bar = 1 μm.

Environmental experiences, such as social isolation, can alter myelination in developing and adult mice (Liu et al., 2012; Makinodan et al., 2012). Previously, we found that normally developing mice housed in EE from P15 to P45 did not display significant anatomical alterations in subcortical WM myelination (Forbes et al., 2020). However, our OL-specific sequencing indicates that dramatic molecular changes related to myelin outgrowth occur after protracted periods of EE. To determine if additional time in EE translated to ultrastructural changes in myelination, we performed electron microscopic analysis of the corpus callosum (CC; Figure 2A) on mice continuously housed in EE from P15 until either P60 or P90 (Figure 2B). At P60, there was no difference in the number of myelinated axons in the CC (Figures 2C,F). However, myelin thickness was significantly increased (lower g-ratio), indicative of faster conduction velocity (Figures 2D–F). These data indicate that after 45 days of continuous EE, subcortical WM OLs produce additional myelin sheath for existing internodes, without increasing the number or length of internodes. Interestingly, by P90, EE increased both the number of myelinated axons as well as myelin thickness (Figures 2G–J). These data are indicative of a gradual OL response that begins with the thickening of myelin sheaths, followed by either a lengthening of existing internodes or an increase in the number of internodes.

### Prolonged EE Alters OL Lineage Cell Dynamics in the Subcortical WM

**FIGURE 3:**
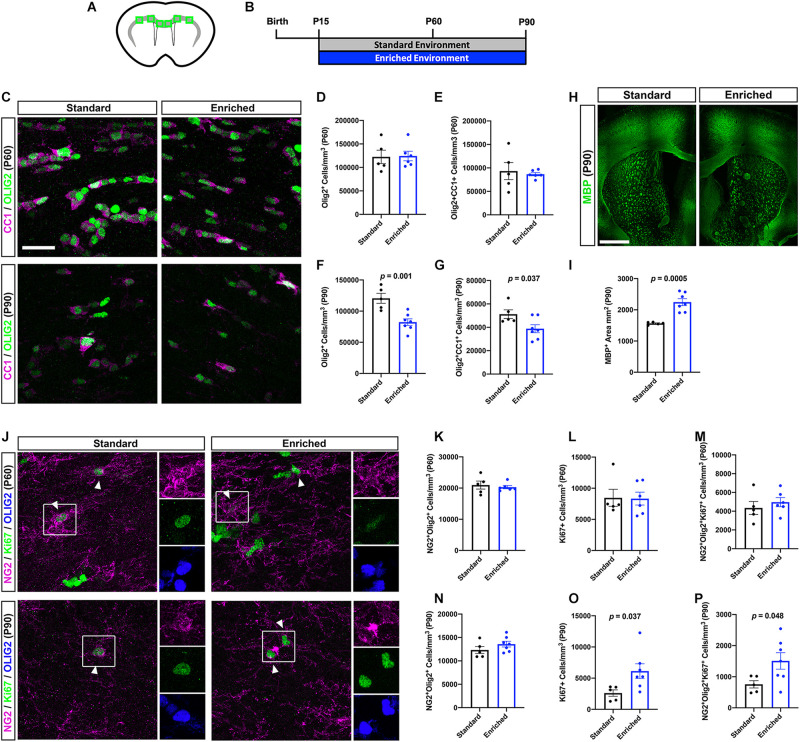
Prolonged EE alters OL lineage cell dynamics in the subcortical WM. **(A)** Schematic depiction of the subcortical WM (gray) in a coronal slice, with green boxes representing the quantified regions (corpus callosum, cingulum, external capsule). **(B)** Experimental timeline. **(C)** Representative confocal images of CC1 (magenta) and OLIG2 (green)-expressing OLs in the subcortical WM at P60 (top) and P90 (bottom). **(D,F)** Quantification of OLIG^+^ OL lineage cells in the subcortical WM of SD (black) and EE (blue) mice at P60 **(D)** and P90 **(F)**. **(E,G)** Quantification of OLIG2^+^/CC1^+^ OLs at P60 **(E)** and P90 **(G)**. **(H)** Representative images of coronal slices immunohistochemically labeled with MBP (green) at P90. **(I)** Quantification of MBP^+^ area in the subcortical WM at P90. **(J)** Representative confocal images of NG2 (magenta) and Ki67 (green)-expressing OPCs in the subcortical WM at P60 (top) and P90 (bottom). Arrowheads demarcate proliferating OPCs. **(K,N)** Quantification of NG2^+^/OLIG2^+^ OPCs at P60 **(K)** and P90 **(N)**. **(L,O)** Quantification of Ki67^+^ proliferating cells at P60 **(L)** and P90 **(O)**. **(M,P)** Quantification of NG2^+^/OLIG2^+^/Ki67^+^ proliferating OPCs at P60 **(M)** and P90 **(P)**. Scale bar = 1mm for panel **(H)** and 25 μm for all other images.

Based on the ultrastructural changes in myelination observed after prolonged EE, we next sought to characterize OL lineage cell dynamics in the subcortical WM (Figures 3A,B). Because our previous work did not find significant differences in OL lineage cells after EE from P15 to P45 (Forbes et al., 2020), we again chose to examine the P60 and P90 timepoints. First, we quantified OLIG2^+^ OL lineage cells, and OLIG2^+^CC1^+^ post-mitotic OLs in the subcortical WM. At P60, prolonged EE did not alter the density of OL lineage cells or differentiated OLs (Figures 3C–E). Interestingly, at P90, the density of OL lineage cells and OLs was significantly decreased in mice housed in EE (Figures 3C,F–G). However, this decrease in OL density was concomitant with an increase in subcortical WM area, as determined by myelin basic protein (MBP) expression (Figures 3H–I). These data indicate that after prolonged periods of EE, increased oligodendrogenesis is not responsible for increases in myelination.

We next asked whether OL progenitor cell (OPC) proliferation was impacted by prolonged EE. Indeed, OPCs respond to a variety of environmental stimuli—such as neuronal activity or injury—by proliferating (Gibson et al., 2014; Hesp et al., 2015; Mitew et al., 2018; Adams et al., 2020). At P60, we found no significant changes in Ki67^+^ proliferating cells, or NG2^+^OLIG2^+^Ki67^+^ proliferating OPCs (Figures 3J–M), indicating that OL lineage cell numbers are not altered after 45 days of EE, despite myelin thickening. Alternatively, at P90 EE caused a significant increase in proliferating cells and OPCs in the subcortical WM (Figures 3J, N–P). This increase in new OPCs is likely either in response to elevated neural stimulation, or to the reduction in OLs observed at this timepoint. Importantly, EE does not alter expression of IBA1, indicating that the EE-induced changes in OL lineage cell dynamics occur independently of influence from microglia (Supplementary Figure 1).

### Prolonged EE Improves Subcortical WM-Dependent Locomotor Coordination

**FIGURE 4:**
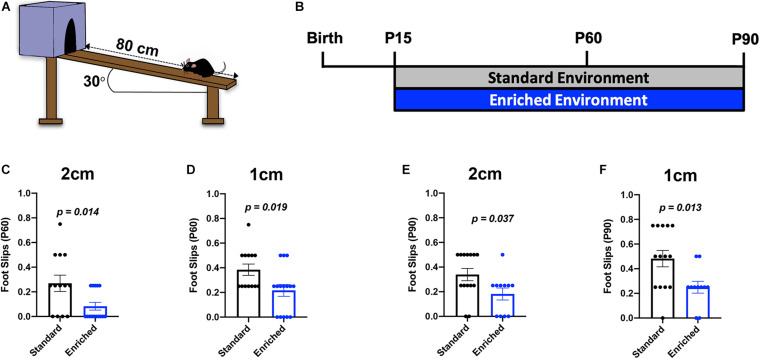
Prolonged EE improves locomotor coordination. **(A)** Schematic depiction of the inclined beam-walking task. **(B)** Experimental timeline. **(C)** Quantification of foot slips for the 2-cm beam at P60. **(D)** Quantification of foot slips for the 1-cm beam at P60. **(E)** Quantification of foot slips for the 2-cm beam at P90. **(F)** Quantification of foot slips for the 1-cm beam at P90.

Previous studies determined that deficits in subcortical WM-dependent locomotor coordination following hypoxic brain injury are partially recovered with improved myelination (Scafidi et al., 2014; Forbes et al., 2020). To determine if the prolonged EE-induced changes in subcortical WM myelination translated into functional improvements in locomotor coordination, we utilized the inclined beam-walking task at P60 and P90 (Figures 4A,B). This behavioral assessment challenges mice to traverse a wooden beam at a 30^o^ incline, while the number of foot slips is counted. Previously, we showed that normally developing mice housed in EE from P15 to P45 did not show significant improvement in locomotor coordination, despite a moderate decrease in foot slips (Forbes et al., 2020). Here, we found that EE mice had significantly fewer foot slips than SD mice on both the 2 cm-wide beam and the increasingly difficult 1 cm-wide beam at P60 (Figures 4C,D). These data indicate that thicker subcortical WM myelin is sufficient to improve locomotor coordination. Expectedly, mice housed in EE until P90 also had significantly fewer foot slips on both beams (Figures 4E,F). Consistent with previous findings, sexually dimorphic changes in locomotor coordination were not observed in these mice (Forbes et al., 2020; Supplementary Figure 2). Together, these data show that protracted periods of environmental experiences are capable of improving motor performance by altering subcortical WM myelination.

## Discussion

Myelin provides structural and metabolic support to axons, while also increasing saltatory conduction (Nave and Werner, 2014). The ability to provide fast and accurate axonal conduction is essential to motor, sensory, and cognitive abilities. Thus, disturbances in myelin damages neurological circuit function and causes devastating behavioral impairments. Interestingly, environmental stimuli have profound effects on myelination that improve circuit and behavioral function. For example, piano playing, juggling, and learning a second language are all associated with structural alterations in WM (Bengtsson et al., 2005; Scholz et al., 2009; Hosoda et al., 2013). Therefore, a better understanding of how experiential stimuli can modulate myelination has great potential for promoting myelin repair.

Environmental enrichment has been widely used to study experiential-dependent effects on the brain. Anatomically, EE increases cortical thickness, dendritic arborization, and spine density (reviewed in Sale et al., 2009). Additionally, EE increases hippocampal neurogenesis, which we previously confirmed using our paradigm (Kempermann et al., 1997; Forbes et al., 2020). These EE-induced alterations promote learning and memory, and ameliorate age-, disease-, and injury-related functional loss (reviewed in van Praag et al., 2000). However, in the context of OL lineage cells and myelination, EE has been disproportionately studied in adult animals. For example, EE increases OPC proliferation after demyelination and in uninjured adult mice, while also reducing age-related myelin loss in the corpus callosum (Magalon et al., 2007; Ehninger et al., 2011; Zhao et al., 2012; Yang et al., 2013). Our previous work addressed this gap in knowledge by asking whether EE could promote recovery from developmental brain injury (Forbes et al., 2020). We demonstrated that, after neonatal hypoxia, continuous EE from P15-P45 promoted oligodendrogenesis, increased myelination, and restored subcortical WM-dependent locomotor coordination. These exciting findings revealed that EE could be harnessed to impact myelination during a critical period of intervention.

Presently, in a non-pathological setting, we show that 30 days of EE (from P15-P45) positively influence molecular processes related to synapse potentiation, cellular outgrowth, and calcium signaling. In OLs, all of these processes are attributable to myelin extension and wrapping (Waggener et al., 2013; Hughes and Appel, 2019). To an extent, these alterations mimic the previously observed EE-induced changes after perinatal hypoxia, including increased expression of genes related to cell morphology and outgrowth (Forbes et al., 2020). However, we previously found that EE also reverses the hypoxia-induced decrease in OL lipid metabolism, whereas in the non-pathological setting, EE doesn’t significantly impact lipid metabolism. Thus, EE-dependent mechanisms of developmental myelination diverge from mechanisms promoting myelin repair after developmental injury.

Despite substantial changes to the OL translatome, we knew from previous analyses that 30 days of EE had no significant effects on subcortical WM myelination (Forbes et al., 2020). However, a trend toward improved locomotor coordination suggested that modest changes could be occurring after 30 days of EE exposure, and we hypothesized that additional time in EE would lead to significant anatomical and behavioral changes. Indeed, this hypothesis proved correct as mice housed continuously from P15 until either P60 or P90 displayed significant alterations in myelin ultrastructure. At both timepoints examined, the myelin sheaths were thicker, indicative of faster conduction velocity. Similar increases in myelination have been reported previously, either by genetic manipulation of OLs or after neuronal activation (Flores et al., 2008; Gibson et al., 2014; Jeffries et al., 2016; Mitew et al., 2018). Interestingly, direct manipulation of OLs through constitutive activation of Akt1 or ERK1/2, produces thicker myelin, despite no significant changes in oligodendrogenesis (Flores et al., 2008; Jeffries et al., 2016). In line with these findings, EE-induced myelination did not increase OL density or oligodendrogenesis, suggesting that existing OLs extend additional myelin sheaths resulting in reduced g-ratio, and increased WM area. This is supported by recent discoveries that pre-existing OLs extend myelin sheaths in response to sensory manipulation or during recovery from a demyelinating insult (Duncan et al., 2018; Bacmeister et al., 2020; Yang et al., 2020). However, another possibility is that OLs generated after mice were housed in EE are capable of generating thicker and more sheaths.

Regardless of whether increased myelination stems from new or existing oligodendrocytes, an important question to answer is whether behavioral functions are improved. Following optogenetic stimulation of premotor cortical neurons and thickened myelin in the corpus callosum, gait analysis of mice revealed increased limb swing speed (Gibson et al., 2014). Furthermore, hypermyelination due to constitutively-expressed ERK1/2 in OLs enhanced hippocampal-dependent associative emotional memory formation, as measured by a fear conditioning test (Jeffries et al., 2016). In the present study, we found that prolonged EE-induced increase in corpus callosum myelin thickness translated into improved performance during a subcortical WM-dependent locomotor coordination task. Further mechanistic understanding of each of these unique model systems and experimental paradigms that promote myelination and functional improvements will be essential to develop targeted treatments for myelin repair.

A further important aspect of our work is that EE consists of multiple components—physical activity, novel objects, and socialization—that synergize to induce significant changes in developmental myelination under pathological conditions (Forbes et al., 2020). However, in the present study, we did not investigate whether individual components of EE are sufficient to induce similar effects under normal physiological conditions. Previous research suggests that these individual components may have significant effects on myelination. For example, adult or juvenile mice kept in social isolation have decreased myelination in the prefrontal cortex and impaired social behavior (Liu et al., 2012; Makinodan et al., 2012). In the case of physical activity, people with higher aerobic fitness levels tend to have higher fractional anisotropy in WM regions, a neuroimaging readout of myelin ultrastructure (Oberlin et al., 2016). Additionally, mice with access to voluntary exercise have increased rates of oligodendrogenesis in the brain and spinal cord (Krityakiarana et al., 2010; Simon et al., 2011). Conversely, we recently found that EE-induced recovery from perinatal brain injury was only effective if all components of EE were included. This evidence, combined with the requirement of prolonged exposure, suggests that all components of EE may be necessary to induce significant changes in developmental myelination. Nevertheless, future work should determine the individual contributions of each EE component during normal developmental myelination.

Overall, the effects of EE on neuroplasticity and neurological recovery are well described. Here, we show that prolonged exposure to subtle environmental experiences causes molecular changes in OLs, and has a profound impact on developmental myelination and behavioral function. Because of the protracted timeline of myelination in the CNS, exogenous stimuli have great potential for functional impact. Therefore, leveraging mechanistic insight into experience-dependent myelination will inform future treatments and/or prevention of neurological disease.

## Data Availability Statement

The original contributions presented in the study are publicly available. This data can be found here: Sequence Read Archive (SRA), BioProject ID PRJNA716661.

## Ethics Statement

The animal study was reviewed and approved by Institutional Animal Care and Use Committee (IACUC) of Children’s National Hospital.

## Author Contributions

EG, TF, and VG designed and conceptualized all the experiments. EG, VP, and TF performed and analyzed all the experiments in this project with the exception of Electron Microscopy, performed by JD. EG wrote the manuscript, with input from VP and VG. All authors contributed to the article and approved the submitted version.

## Conflict of Interest

The authors declare that the research was conducted in the absence of any commercial or financial relationships that could be construed as a potential conflict of interest.

## Supplementary Material

Supplementary Table 1 Top 50 OL RNAs from P22 subcortical WM.

Supplementary Table 2 Top 50 OL RNAs from P30 subcortical WM.

Supplementary Table 3 Top 50 OL RNAs from P45 subcortical WM.

Supplementary Table 4 Differentially expressed genes between EE and SD at P22.

Supplementary Table 5 Differentially expressed genes between EE and SD at P30.

Supplementary Table 6 Differentially expressed genes between EE and SD at P45.
