# Supplementary material for: Prolonged Environmental Enrichment Promotes Developmental Myelination
Source: Front Cell Dev Biol. 2021 Apr 26;9:665409. doi: 10.3389/fcell.2021.665409 (PMC8107367; doi:10.3389/fcell.2021.665409)
Supplement: Supplementary file 7 [file Table_7.DOCX]

Supplementary Material


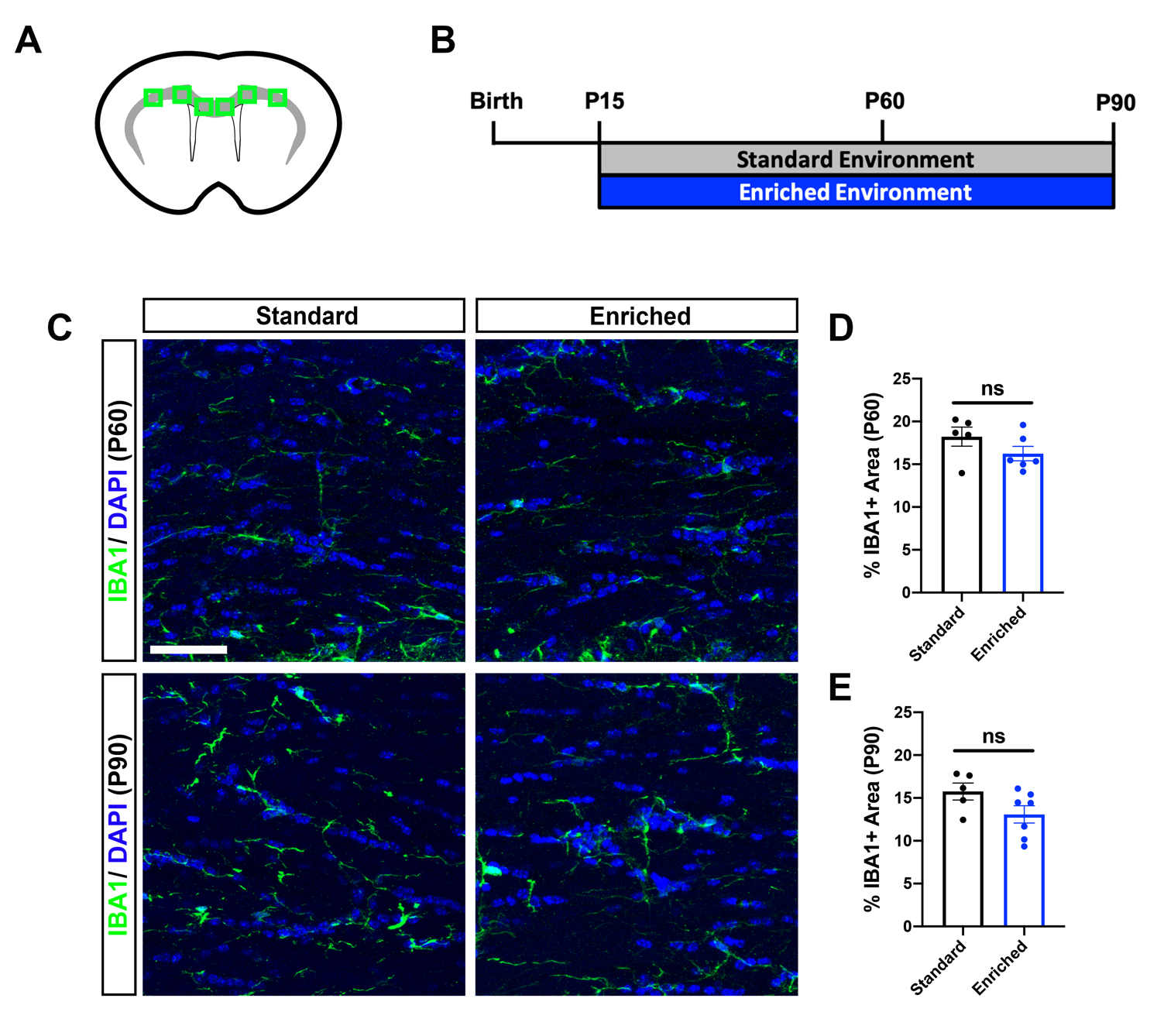


**Supplementary Figure 1.** Prolonged EE has no effect on microglia in the subcortical WM. **(A)** Schematic depiction of the subcortical WM (grey) in a coronal slice, with green boxes representing the quantified regions (corpus callosum, cingulum, external capsule). **(B)** Experimental timeline. **(C)** Representative confocal images of IBA1 (green) and DAPI (blue)-expressing microglia in the subcortical WM at P60 (top) and P90 (bottom). **(D-E)** Quantification of the percentage of IBA1+ area in the subcortical WM of SD (black) and EE (blue) mice at P60 (D) and P90 (F). Scale bar = 50μm


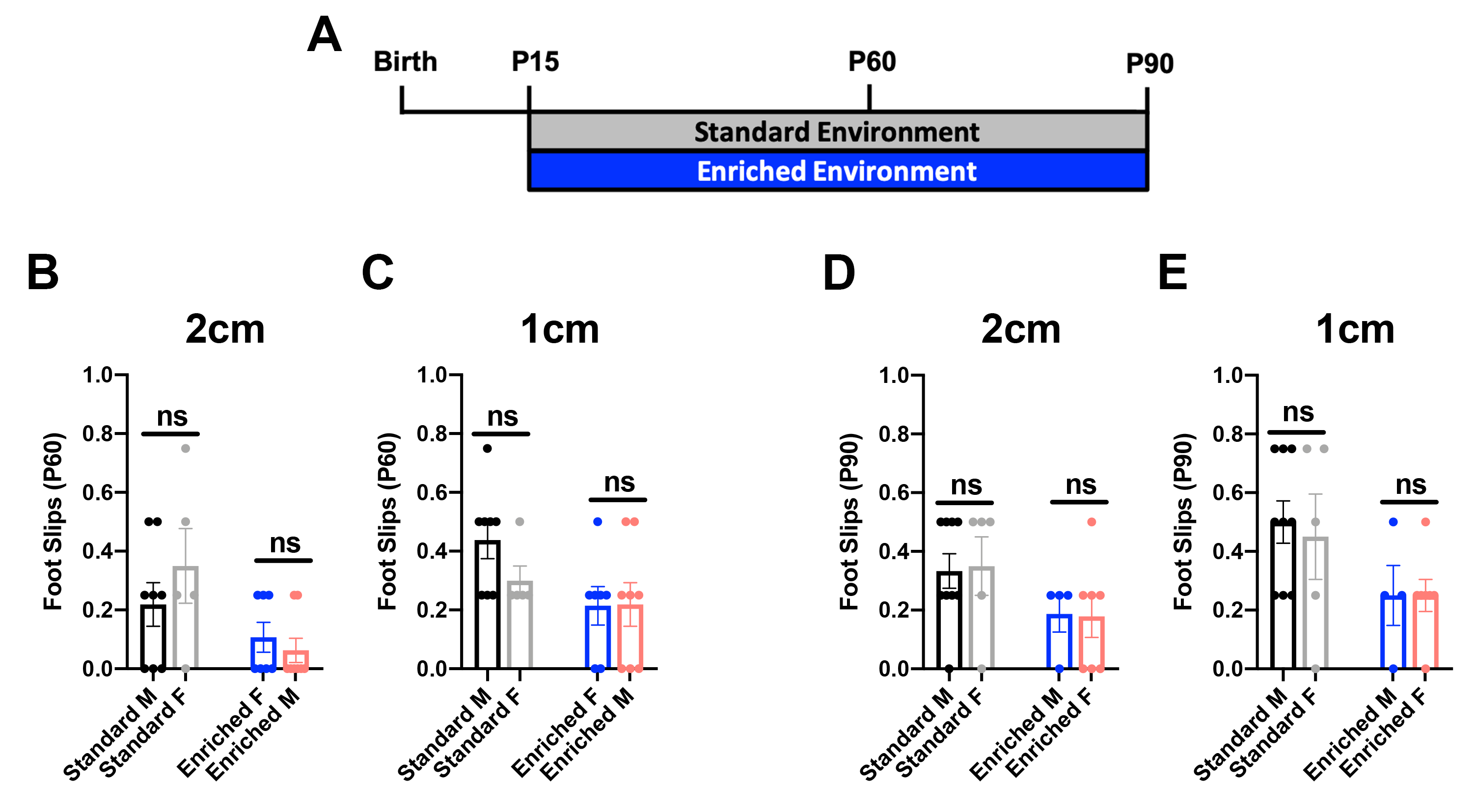


**Supplementary Figure 2.** Sex-specific differences in locomotor coordination are not evident following prolonged EE. **(A)** Experimental timeline. **(B)** Quantification of foot slips for male (M) and female (F) mice on the 2-cm beam at P60. **(C)** Quantification of foot slips for the 1-cm beam at P60. **(D)** Quantification of foot slips for the 2-cm beam at P90. **(E)** Quantification of foot slips for the 1-cm beam at P90.
